# STERCORAL COLITIS DUE TO PICA SYNDROME IN A YOUNG ADULT: A CASE REPORT

**DOI:** 10.51894/001c.123079

**Published:** 2024-08-30

**Authors:** Anh T. Mai

**Affiliations:** 1 Internal Medicine Detroit Medical Center Sinai-Grace Hospital

## 75

### INTRODUCTION

Stercoral colitis is the inflammation of the large bowel wall caused by the increased intraluminal pressure secondary to fecal impaction. This rare condition tends to occur in the elderly with chronic constipation, which will develop into fecalomas. This can lead to severe complications, including ischemic colitis, ulceration, and perforation. Most cases are seen in elderly patients being bedbound due to dementia or stroke or patients with chronic opioid use. It may be seen in younger patients with psychiatric conditions, though much less frequently, resulting in a gap in knowledge regarding the presentations and outcomes of these patients. We report a young adult with sepsis secondary to stercoral colitis due to PICA syndrome.

### CASE DESCRIPTION

A 20-year-old male with a history of severe non-verbal autism spectrum disorder presented to the emergency department with abdominal pain, poor oral intake, and bloody diarrhea for 5 days. On admission, he presented with fever, marked abdominal distension, tachycardia, and tenderness. Laboratory findings were significant for hemoglobin of 5.4 g/dl, leukocytosis, and thrombocytosis. Stercoral colitis was established based on a CT scan of the abdomen, which illustrated severe fecal impaction in the rectosigmoid colon, distending the rectum approximately 9.5 cm in axial radius, and extending craniocaudally 25 cm, together with circumferential rectosigmoid wall thickening with mild scattered serosal stranding. The patient was managed with fluid resuscitation, intravenous antibiotics, and total parenteral nutrition. After a gastrografin enema, manual disimpaction was performed in the operating room followed by a sigmoidoscopy. Pathology results revealed a non-organic appearing rectal foreign body which was identified as a large mass of fecal material mixed with red and green thread-like material, hair, tan-gray mesh, unidentifiable string-like material and an object with metallic appearance. The patient was able to resume oral nutrition and discharged after his condition improved.

### DISCUSSION

Although rare, stercoral colitis can occur in young adults with neurologic or psychiatric comorbidities. Since initial manifestations may be varied, and mortality associated with complications such as perforation and septic shock can be as high as 60%, special attention should be paid to prompt diagnosis and effective management to avoid the risk of progression.

